# Prevalence of Vitamin D Deficiency in Sickle Cell Disease: A Systematic Review

**DOI:** 10.1371/journal.pone.0119908

**Published:** 2015-03-03

**Authors:** Vikki G. Nolan, Kerri A. Nottage, Elliott W. Cole, Jane S. Hankins, James G. Gurney

**Affiliations:** 1 Departments of Hematology, Division of Epidemiology, Biostatistics and Environmental Health, University of Memphis, School of Public Health, Memphis, TN, United States of America; 2 Epidemiology and Cancer Control, Division of Epidemiology, Biostatistics and Environmental Health, University of Memphis, School of Public Health, Memphis, TN, United States of America; 3 St. Jude Children’s Research Hospital, Memphis, TN, United States of America; Sickle Cell Unit, JAMAICA

## Abstract

Vitamin D deficiency has emerged as a public health focus in recent years and patients with sickle cell disease (SCD) reportedly have a high prevalence of the condition. Our objectives were to summarize definitions of vitamin D deficiency and insufficiency used in the literature, and to determine the prevalence and magnitude of each in patients with SCD through a systematic review conducted according to PRISMA guidelines. From a PubMed search, 34 potential articles were identified and 15 met eligibility criteria for inclusion. Definitions of deficiency and insufficiency varied greatly across studies making direct comparisons difficult. This review provides evidence to suggest that suboptimal vitamin D levels are highly prevalent among those with SCD, far more so than in comparable non-SCD patients or matched control populations. Defining deficiency as vitamin D <20ng/mL, prevalence estimates in SCD populations range from 56.4% to 96.4%. When compared with results from the population-based National Health and Nutrition Examination Survey, however, the general African American population appeared to have a similarly high prevalence of vitamin D deficiency. African American patients with and without SCD were both substantially higher than that of Caucasians. What remains to be determined is whether there are adverse health effects for patients with SCD because of concurrent vitamin D deficiency.

## Introduction

### Rationale

Vitamin D (25-hydroxyvitamin D) deficiency has emerged as a public health focus in recent years for its contribution to adverse skeletal and extra-skeletal manifestations[1]. Moreover, individuals living with sickle cell disease (SCD) reportedly have a high prevalence of vitamin D deficiency [2]. Race, age, body mass index (BMI), latitude, diet, sunlight exposure, and skin pigmentation are all factors influencing vitamin D status [3,4]. In addition to its effects on bone health [5,6], vitamin D deficiency has been linked to multiple health conditions including cardiovascular disease [7–9], asthma [10–12], nephropathy [1,13], and chronic pain [14–19]. Patients with SCD are susceptible to all of these complications [20–24], although it is unclear to what extent vitamin D deficiency is a contributing causal factor.

Vitamin D metabolism is complex and requires homeostasis among several different organ systems, including the skin, intestines, liver, kidney, and parathyroid [23]. Vitamin D deficiency is now recognized as one of the most common nutritional conditions among persons with SCD [2,25,26] and there are characteristics specific to SCD that may contribute to this phenomenon. First, those with SCD may experience decreased appetite [27,28] or be unable to adequately absorb nutrients due to damage to the intestinal mucosa [29,30]. Second, due to the constant production of red blood cells to compensate for shortened red blood cell survival, those with SCD have an increased basal metabolic rate with higher nutritional demands to sustain normal physiologic functioning [31–39]. It has been reported that 80% of African Americans have some degree of lactose intolerance [40] and therefore may avoid vitamin D rich foods, which suggests that those with SCD may not be meeting these increased nutritional demands. Aside from nutritional causes, persons with SCD may have renal impairment secondary to their hematological disease which may impede conversion of vitamin D to its active form. Many, if not most, SCD patients in the U.S. are dark skinned with resultant decreased ability to synthesize vitamin D from sunlight. Finally, SCD is an inflammatory condition and vitamin D binding protein has been shown to decline in inflammatory conditions [41]. The motivation for better understanding the magnitude of vitamin D deficiency among populations with SCD, and whether the prevalence is higher among those with SCD compared with similar persons without SCD, is that vitamin D deficiency can be reliably and inexpensively treated, making it a prime intervention to potentially improve health outcomes.

### Objectives

Among SCD populations, the prevalence of vitamin D deficiency and insufficiency has been described in both children and adults, although study groups are often heterogeneous, definitions are inconsistent, and most studies are uncontrolled in that they do not compare prevalence to similar populations without SCD. Our objectives were to identify studies that have assessed the prevalence of vitamin D deficiency in patients with SCD, summarize definitions of deficiency and insufficiency used in the literature, and to determine whether the magnitude of vitamin D deficiency and insufficiency in patients with SCD differs from that of comparable populations without SCD.

## Methods

This review was conducted according to the guidelines outlined in Preferred Reporting Items for Systematic Reviews and Meta-Analyses: The (PRISMA) Statement[42].

### Eligibility Criteria

Manuscripts considered eligible for this review included only those: 1) written in English, 2) original articles published as of July 1, 2014, 3) observational or experimental studies (i.e. abstracts, review articles, and case-reports or case-series were not included).

### Information Sources and Search Strategy

The Medline bibliographic database of the National Library of Medicine, accessed through the National Institutes of Health’s PubMed online resource, was searched for articles to include in this review. The primary search included the Medical Subject Headings (MeSH) keywords “sickle cell disease” AND “vitamin D” AND “deficiency”. The search was repeated using only “sickle cell disease” AND “vitamin D” to ensure no relevant articles were missed. Additionally, articles referenced by those identified in this search were reviewed for relevance.

### Study Selection

The abstracts and keywords were examined to ensure that the articles met eligibility criteria. Articles were then reviewed to determine if vitamin D deficiency was assessed in the study population. Experimental studies were included only if the authors assessed vitamin D deficiency at baseline, or before any treatment began. Articles in which study subjects were recruited based on their vitamin D status were excluded from the review.

### Data Collection Process and Data Items

For each study that was determined to be eligible, the number and ages of subjects, presence or lack of a comparison group, definitions of vitamin D deficiency and/or insufficiency, and the prevalence of each condition were abstracted (S1 Table). Additional findings related to secondary aims were also reported in the results of individual studies.

## Results

### Study Selection

**Fig 1:**
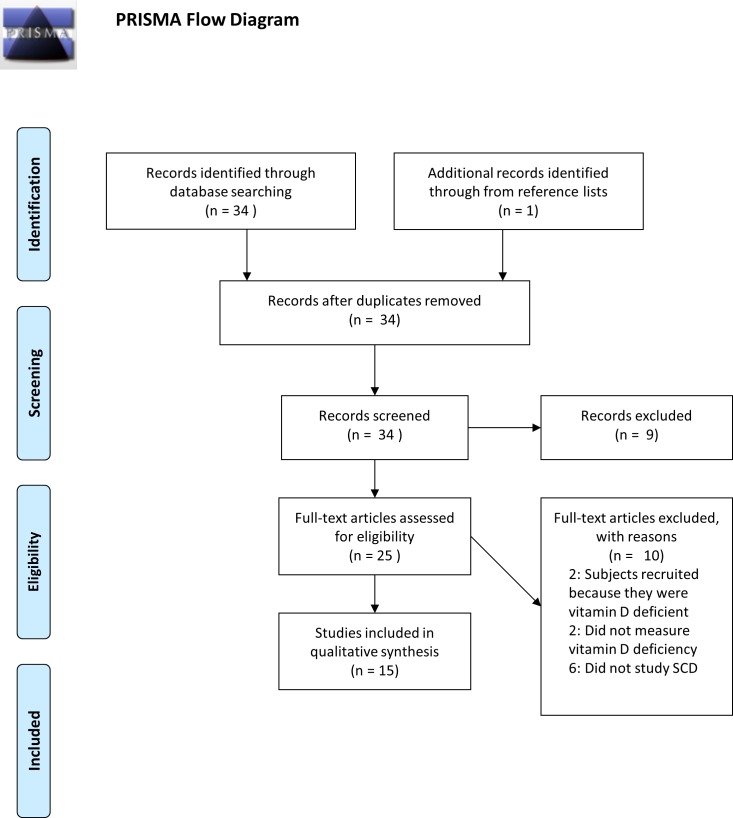
PRISMA Flow Diagram. Flowchart of publications included in this systematic review.

After duplicates were removed, 33 articles were identified using the MeSH keywords previously described. Not eligible for review included two articles published in languages other than English, four case-reports or case-series, and three review articles. Additionally, two articles studied vitamin D levels in their study populations but did not report the prevalence of deficiency, and in two studies participants were recruited specifically because they were vitamin D deficient. Finally, there were six articles that were not applicable in that “vitamin D” and “sickle cell disease” are mentioned in the abstract, however the primary study population did not consist solely of subjects with sickle cell disease. One additional article published by Miller et al. [43], was included in the review because it was referenced by five of the articles already identified for inclusion in the review and met eligibility criteria. This article was missed by our keyword search since “vitamin D” was not one of the MeSH keywords associated with this manuscript. In all, 15 studies were included that quantified the prevalence of vitamin D deficiency in the SCD population (Fig. 1).

### Study Characteristics

More than half of the included studies (8/15) were conducted in the United States[2,43–49]. The remaining studies were conducted in Turkey [50], Curaco [51], Saudi Arabia [52,53], Spain [26] or France[25,54]. In 10 studies, the primary aim of the paper was to assess the vitamin D status of the participants[2,25,26,44,45,49–53]. The primary aim in the remaining studies was to explore the association between vitamin D status and clinical outcomes such as bone mineral density (BMD) [43,46,54] and chronic pain [47,48]. Most of the articles included in this review (n = 11/15) studied pediatric/adolescent populations [2,26,45–52,54] and only four included a non-sickle cell comparison group [2,49,52,53]. Four studies assessed seasonal variation[2,25,44,45] and three studied dietary intake of vitamin D [2,49,52]. Definitions of vitamin D deficiency and insufficiency varied greatly in these studies.

### Results of Individual Studies

In 10 studies, the primary aim of the paper was to assess the vitamin D status of the participants; six of those studied pediatric populations and three of those were conducted in the United States. The first, by Buison et al. [2], assessed vitamin D status, dietary intake of vitamin D, and seasonal variation in 65 children with homozygous SS disease (HbSS), aged 5 to 18 years, recruited from a longitudinal study of growth and nutrition at the Comprehensive Sickle Cell Center at Children’s Hospital Philadelphia (CHOP). Researchers compared the prevalence of vitamin D deficiency in those with HbSS to that in a group of normal, healthy African American (n = 33) and non-African American (n = 76) children aged 7 to 10 years participating in a bone health and nutrition study at CHOP[2]. Vitamin D status was dichotomized; low vitamin D was defined as <27.5 nmol/L (equivalent to <11ng/mL) and normal ≥27.5 nmol/L. They found that children with HbSS had significantly lower serum vitamin D concentrations than healthy children and that the prevalence of low vitamin D was substantially higher in those with HbSS (65%) compared with healthy African American (6%) and non-African American children (0%). Dietary intake of vitamin D was assessed using a 24-hour recall. Low dietary intake of vitamin D was significantly associated with lower serum vitamin D levels in both children with HbSS and healthy children.

The second study of children in the US by Rovner et al. [49] assessed 61 children ages 5 to 18 years with HbSS disease and 89 healthy African-American controls ages 6 to 18 years. Vitamin D deficiency, as in Buisson et al., was defined as <11ng/mL and insufficiency as 11 to <30 ng/mL. Twenty participants (33%) with HbSS were deficient, compared with only 9% of healthy children. Proportions of vitamin D insufficiency were found to be similar in the two groups: 93% of those with SCD and 90% of the healthy children. Finally, Rovner et al. [49] found that adjusted for season and age, those with SCD were 5.3 (95% CI: 2.5, 8.2) times more likely to be vitamin D deficient than were healthy controls. A report by Jackson et al. [45] examined vitamin D deficiency and comorbidities in children with SCD. One hundred and thirty-nine pediatric patients with HbSS were enrolled. Severe deficiency was defined as ≤10ng/mL, deficiency was defined as >10 to ≤20ng/mL, and insufficiency was defined as >20 to <30ng/mL. The authors found that 96% were deficient and, of these, 64% had severe deficiency. Only 2% of study subjects had normal vitamin D levels. Though the participants were deficient all year round, vitamin D levels varied by season with the lowest levels in winter and highest levels in summer. The secondary aim of the Jackson study [45] was to determine the association between Vitamin D levels and comorbidities. Severe vitamin D deficiency was associated with age and decreased lung function but not pain episodes, acute chest syndrome episodes, asthma or body mass index.

Five international studies sought to determine the prevalence of vitamin D deficiency in children with SCD. In a study of vitamin D status in children living in Madrid, Spain, Garrido et al. [26] assessed 78 children, ages 0 to 16. SCD genotypes included HbSS as well as hemoglobin SC disease (HbSC), sickle β-zero (HbSβ^0^) thalassemia, and sickle β-plus (HbS^+^ (Sβ^+^) thalassemia. Deficiency was defined as <20ng/nL, borderline 21 to ≤30ng/mL and normal >30ng/mL. Nearly 80% were below 30ng/mL and 56.4% were considered deficient. No seasonal variation in vitamin D status was found. Ozen et al. [50] studied 50 children and adolescents (ages 4 to 18) in Turkey; 30 children were HbSS and 20 were compound heterozygote for a β-thalassemia mutation (type not specified). Ozen et al. [50] also defined deficiency as <20ng/nL (<50nmol/L), insufficient as 21 to ≤30ng/mL (50 to <75 nmol/L) and normal >30ng/mL (≥75nmol/L). The authors found that 63.1% were deficient and another 18.4% were insufficient, i.e. 81.5% had vitamin D levels <30ng/mL. Vitamin D levels were significantly lower in children whose height and/or weight were more than 2 standard deviations below the mean. A study by Mohammed et al. assessed vitamin D status in 99 children with HbSS and 104 healthy controls in Saudi Arabia [52]. Participants ranged in age from five to 25 years. They found that vitamin D levels were significantly lower in participants with SCD compared to healthy controls and that 12% of those with SCD were deficient—defined as <10mg/mL—compared with only two percent of health controls. Finally, a study by van der Dijs et al, compared vitamin D levels in 18 children with HbSS (ages 3 to 19) with age-, sex- and ethnicity-matched controls. Using the same definition of deficiency as Mohammed et al. (<25nmol/L or <10ng/mL), they found that none of the study participants had vitamin D levels below this cut point. This is unsurprising given the differences in climate and latitude. A previous reports from Saudi Arabia found low levels of vitamin D due to the avoidance of direct sunlight in the hot, desert climate [55].

Three studies, whose primary aim was to assess vitamin D status in patients with SCD, included only adults; one was conducted in the United States [44], one in France[25], and Saudi Arabia [53]. Goodman et al. enrolled 142 adults with SCD at two clinical centers in the US—Eastern Virginia Medical School (EVMS; n = 84) and University of Chicago (UC; n = 58). The mean age of the participants at EVMS was 31.8 years (range: 21–56) and at UC, the mean age was 34 years (18–63 years). SCD genotypes included were HbSS, HbSC and HbSβ-thalassemia (type not specified) [44]. The definitions of deficiency differed from those used in other studies. Goodman et al. defined severely deficient as <10ng/mL, insufficient as 10 to <16ng/mL, and sub-optimal as 16 to <30 ng/mL. They found that 60% of participants were severely deficient and 100% had sub-optimal levels. Acknowledging the heterogeneity of definitions of vitamin D deficiency, Goodman et al. also determined the proportion of participants with levels of <20ng/mL. They found that 94% of participants would be deficient by this definition.

Sadat-Ali conducted a cross-sectional study of vitamin D levels in adults with HbSS in Saudi Arabia [53]. One hundred sixty-eight participants with SCD and 200 age-, and sex-matched controls were enrolled. The mean age of those with HbSS was 28.4 years ± 4.6 and mean age of the controls was 28.5 years ± 4.1. This study defined deficiency as <20ng/mL and insufficient as 21 to 29 ng/mL. They found that those with SCD had significantly lower vitamin D levels compared to healthy controls. Additionally, they found that 92% of female and 71% of male HbSS participants were deficient, compared with only 11% of healthy females and 10% of males. The last study that sought to determine the prevalence of vitamin D deficiency was conducted by Arlet et al. Fifty-six consecutive adult patients (ages 17 to 67 years; mean 29.6 ± 9.5 years) with SCD (HbSS or HbSC) were enrolled in the study [25]. Deficiency was defined as <10 ng/mL and insufficiency defined as 10 to <30 ng/mL. Arlet et al. found that 75% were deficient and 25% were insufficient; i.e. none had normal vitamin D levels. Arlet et al. also found seasonal variation in vitamin D levels.

The final five studies had primary aims that did not include assessing the prevalence of vitamin D deficiency; instead the authors were studying the association of vitamin D levels with a clinical outcome such as BMD [43,46,54] and chronic pain [47] or conducting a clinical trial of vitamin D supplementation [48]. A report by Osunkwo et al., evaluated vitamin D levels in 53 pediatric patients, ages 1 to 19 years, to determine its association with chronic pain [47]. The cut points used to categorize vitamin D levels were unlike those used in any of the previously mentioned studies. Insufficiency was defined as <75 nmol/L (<30 ng/mL), deficiency was defined as 30 to 50 nmol/L (12 to 20 ng/mL), severe deficiency was defined as 15 to 27.5 nmol/L (6 to 11 ng/mL), and profound deficiency was defined as <12.5 nmol/L (< 5 ng/mL). They found that 15% were insufficient, 32% were deficient, 40% were severely deficient and 13% were profoundly deficient; none had normal vitamin D levels. They also found that both chronic pain and bone fragility, as defined by radiographic evidence of avascular necrosis and/or vertebral compression fractures, were both associated with lower levels of vitamin D. Building on this work, Osunkwo et al. subsequently conducted a pilot randomized clinical trial of oral, high-dose vitamin D to determine its efficacy in reducing pain [48]. Forty-six subjects (ages 7 to 21 years) were enrolled and 39 were included in the final analyses. SCD genotypes included HbSS, HbSC, HbSβ^0^- and Hbβ^+^-thalassemia.HbSβ^+^-thalasemmia. At baseline, 82.5% of participants were insufficient (<75 nmol/L; <30 ng/mL)) for vitamin D and 52.5% were deficient (<50 nmol/L; <20 ng/mL). Among those randomized to supplementation, Osunkwo et al. reported an increase in serum vitamin D levels and improved physical function scores, and a decrease in the number of pain days. While statistically significant, all correlations were modest.

The remaining studies included in this review sought to determine risk factors for low BMD among patients with SCD. Chapelon et al studied 53 pediatric patients aged 9 to 19 years in Paris, France. SCD genotypes included HbSS, HbSC and HbSβ-thalassemia [54]. Deficiency was defined as <12 ng/mL (30nmol/L) and was found in 76% of patients. Despite this high prevalence of vitamin D deficiency, no association with low BMD was observed. Miller et al. found similar results in a cross-sectional study of adult SCD participants aged 18 to 51 years. SCD genotypes included HbSS, HbSC and HbSβ-thalassemia (type not specified) [43]. They found that 84% of adults had vitamin D levels of <20 ng/mL and 66% had levels below 10 ng/mL; however, there was no association between abnormally low vitamin D levels and low BMD. Finally, in a study conducted by Lal et al., 25 children, aged 10 to 19 years with HbSS, were enrolled to study BMD and poor bone mineralization [46]. All of the study participants had low vitamin D levels (<62.5nM; 25 ng/mL), 74% had levels below 50nM (20 ng/mL), and 30% had levels below 27.5nM (11.0 ng/mL). Like Chapelon et al. and Miller et al., no association was found between BMD and vitamin D levels.

## Discussion

Vitamin D deficiency is one of the most common nutritional conditions among persons with SCD [2,25,26] and there are characteristics specific to SCD that may contribute to this phenomenon including decreased appetite [27,28], inability to absorb nutrients due to damage to the intestinal mucosa [29,30], as well as an increased basal metabolic rate and higher nutritional demands to sustain normal physiologic functioning [31–39]. Vitamin D deficiency has been associated with bone health [5,6], cardiovascular disease [7–9], asthma [10–12], nephropathy [1,13], and chronic pain [14–19] and individuals with SCD are susceptible to all of these complications [20–24]. While the role of vitamin D deficiency as a contributing factor in these complications is unclear, vitamin D deficiency can be reliably and inexpensively treated, making it a prime intervention to potentially improve health outcomes among those with SCD.

### Summary of Evidence

This review provides evidence to suggest that vitamin D deficiency is highly prevalent among those with SCD and that the prevalence is higher than that of similar, study-specific comparison populations. When these findings are compared with those from the population-based National Health and Nutrition Examination Survey (NHANES), however, it is less clear whether the increased prevalence of vitamin D deficiency among those with SCD is higher than the general population. A study of trends in vitamin D status found that the prevalence of normal levels (>30ng/mL) among African Americans (ages 12 or older) was only 3% in NHANES 2001–2004 and that the prevalence of severe deficiency <10ng/mL) rose from 9% in NHANES III (1988–1994) to 29% in NHANES 2001–2014 [56]. A more recent report on adult participants of NHANES (2003 through 2006) found that 81% of African American adults were deficient (<20ng/mL) compared with 28% of Caucasians in an analysis of combined NHANES 2003–2004 and 2005–2006 [57]. When looking at the four studies of adults with SCD included in this review [25,43,44,53], only two [43,53] use the same definition of deficiency as Gutierrez et al. [57] and they found the prevalence of vitamin D deficiency to be comparable to the adults in NHANES (82% [53] and 84% [43]). Both Arlet et al. [25] and Goodman et al. [44] reported an extremely high prevalence of vitamin D levels < 30ng/mL, 100% and 98% respectively. Further, Arlet et al. [25] reported that 75% of adults with SCD in their study were below 10 ng/mL and Goodman et al. [44] reported that 86% of their adult participants were below 10 ng/mL. The high prevalence of severe deficiency (<10 ng/mL) in these reports suggest that the prevalence of defiance defined as <20 ng/mL may be even higher than reported by Miller et al. [43] and Sadat-Ali et al. [53], however without the intervening cut point, we are unable to directly compare the results of these studies to the population-based study by Gutierrez et al. [57]

Many mechanisms have been proposed to account for the drastic differences between African Americans and Caucasians in prevalence of vitamin D deficiency/insufficiency. Decreased synthesis of vitamin D in the skin due to increased melanin [58] and differences in diet, particularly avoidance of dairy products, have been suggested as causative factors given that these are the two primary modes of acquiring vitamin D deficiency [34]. It has been reported that 80% of African Americans have some degree of lactose intolerance compared to only 15% of Caucasian Americans, which may account for some proportion of the difference in vitamin D deficiency/insufficiency between the groups [40]. Higher BMI among African Americans have also been implicated as a possible mechanism [59] since body fat has been shown to act as a reservoir for fat-soluble vitamin D and release of the stored vitamin from adipose tissue can be slow [60,61]. It has also been suggested that racial differences in calcium absorption and metabolism may contribute to decreased levels of vitamin D in African Americans. African Americans absorb dietary sources of calcium more efficiently than Caucasians, and are better at retaining calcium in bone and the kidney, especially during growth. These observations suggest that African Americans may need less dietary calcium than Caucasians [62] and thus may also require less vitamin D for calcium metabolism [57,58]. Finally, African Americans may have lower levels of vitamin D binding protein than Caucasian Americans, likely related to racial differences in genetic polymorphisms in vitamin D binding protein genotypes [63]. The clinical consequences of this finding have been the subject of much debate [64], however, they implicate race as an important determinant when interpreting Vitamin D levels. Assessing vitamin D binding protein may be informative in helping to elucidate the bioavailability of vitamin D in SCD, however more research is needed for us to truly understand prevalence of vitamin D deficiency and insufficiency in this population.

To address the definition of “normal,” Wright et al. [65] sought to determine an optimal threshold for vitamin D in African Americans based on its association with intact parathyroid hormone (iPTH). The results of their study found that the level at which iPTH was maximally suppressed was around 20ng/mL in African Americans compared to 30ng/mL in Caucasians, indicating that a lower threshold for defining deficiency in African Americans may be warranted. These findings were in agreement with two other studies that found that iPTH levels in African Americans stabilize around 20ng/mL [57,66].

The studies cited that argue for a lower threshold for vitamin D levels among African Americans included only healthy adults, and excluded those with chronic kidney disease which is known to alter PTH levels and vitamin D metabolism. It is estimated that between 5% to 30% of persons with SCD have decreased kidney function [67,68]. Decreased kidney function, combined with reduced ability to adequately absorb nutrients due to damage to the intestinal mucosa, may drastically affect serum vitamin D levels. This suggests that perhaps neither the threshold for Caucasians nor the threshold suggested for healthy African Americans is applicable to those with SCD. Further research to identify optimal levels of vitamin D among those with SCD is needed.

### Limitations

The reviewed studies have several methodological limitations. First, a majority of these studies have small sample sizes, often clinically-based, limiting the ability to generalize these results to the larger SCD population. Second, several studies assessed vitamin D status at one time point and therefore it is possible that participants may have been misclassified due to seasonal variation. Third, there was variability in the definitions of “deficiency” and “insufficiency” used in these studies making comparisons of the results across studies difficult.

## Conclusions

Notwithstanding the difficulty in comparing across studies because of the variability in classifications of deficiency and insufficiency, this review found vitamin D deficiency to be highly prevalent among patients with SCD in both children and adults. Moreover, several clinically-based studies that compared SCD patients to non- SCD patients or healthy controls showed that vitamin D deficiency is substantially higher among those with SCD disease. When making general comparisons to population-based data in the U.S. from the NHANES studies, however, that conclusion is not as clear. NHANES data shows dramatically higher vitamin D deficiency prevalence in African Americans than in Caucasians, but the prevalence in African Americans is very high generally, and may not differ meaningfully from that of African Americans with SCD. Some research suggests that the threshold for defining adequate versus deficient vitamin D levels, Caucasians may not be appropriate for African Americans with SCD. However, the data that relate vitamin deficiency with poor health outcomes in a causal manner are not robust and, from a preventive and public health perspective, the question of whether there are clear health consequences attributable to vitamin D deficiency (as currently defined) remains to be properly addressed and determined.

## Supporting Information

S1 Table Study Characteristics. (DOCX)
