# Correction: Prevalence of Vitamin D Deficiency in Sickle Cell Disease: A Systematic Review

**DOI:** 10.1371/journal.pone.0128853

**Published:** 2015-05-18

**Authors:** The PLOS ONE Staff

There are errors in the author affiliations. The publisher apologizes for the error. The affiliations should appear as shown here:

Vikki G. Nolan^1^, Kerri A. Nottage^2^, Elliott W. Cole^1^, Jane S. Hankins^2^, James G. Gurney^1,3^

1 Division of Epidemiology, Biostatistics and Environmental Health, School of Public Health, University of Memphis, Memphis, TN, USA, 2 Department of Hematology, St. Jude Children’s Research Hospital, Memphis, TN, USA, 3 Department of Epidemiology and Cancer Control, St. Jude Children’s Research Hospital, Memphis, TN, USA.
