# Supplementary material for: Prevalence of Vitamin D Deficiency in Sickle Cell Disease: A Systematic Review
Source: PLoS One. 2015 Mar 3;10(3):e0119908. doi: 10.1371/journal.pone.0119908 (PMC4347975; doi:10.1371/journal.pone.0119908)
Supplement: S1 Table — (DOCX) [file pone.0119908.s001.docx]

| **Authors** | **Study title** | **Subjects** | **Ages** | **Vitamin D Classification** | **Prevalence** |
| --- | --- | --- | --- | --- | --- |
| Arlet et al. 2013^25^ | Relationship between vitamin D Deficiency and bone fragility in sickle cell disease: A cohort study of 56 adults | n = 56 | 17 to 67 years | Deficiency: <10 ng/mL    Insufficiency: 10 to <30 ng/mL | 75% <10 ng/mL  25% 10 to <30 ng/mL  0% normal |
| Buison et al. 2004^2^ | Low vitamin D status in children with sickle cell disease | n = 65 | 5 to 18 years | Low: <11.2 ng/mL | 65% <11.2 ng/mL |
| Chapelon et al. 2009^54^ | Osteopenia and vitamin D deficiency in children with sickle cell disease | n= 53 | 9 to 19 years | Deficiency: <12 ng/mL | 76% <12 ng/mL |
| Garrido et al.  2012^26^ | Status of vitamin D in children with sickle cell disease living in Madrid, Spain | n = 78 | 0 to 16 years | Deficiency: 20 ng/mL  Borderline: 21 to 29 ng/mL | 56.4% <20 ng/mL  79.5% <30 ng/mL  17.9% <11 ng/mL |
| Goodman et al. 2010^44^ | Prevalence of vitamin D deficiency in adults with sickle cell disease | N = 142 | 21 to 56 years | Severe  deficiency: <10 ng/mL  Insufficiency: <16 ng/mL  Suboptimal: <30 ng/mL | 60% <10 ng/mL  86% <16 ng/mL  98% < 30 ng/mL |
| Jackson et al. 2012^45^ | Vitamin-D deficiency and comorbidities in children with sickle cell disease | N = 139 | 7.9 to 15.1 years | Severely  Deficiency: <10 ng/mL  Deficiency: <20 ng/mL  Insufficiency: 20 to <30 ng/mL | 64% < 10 ng/mL  96.4% <20 ng/mL  1.4% 20 to <30 ng/mL |
| Lal et al. 2006^46^ | Bone mineral density in children with sickle cell anemia | N = 25 | 9 to 19 years | <11 ng/mL  <20 ng/mL  <25 ng/mL | 30% <<11 ng/mL  74% <20 ng/mL    100% <25 ng/mL |
| Miller et al. 2006^43^ | High prevalence and correlates of low bone mineral density in young adults with sickle cell disease | N = 32 | 18 to 51 years | Deficiency: <20 ng/mL | 84% <20 ng/ml  66% <10 ng/mL |
| Mohammed et al. 1993^52^ | Serum calcium, parathyroid hormone and vitamin D status in children and young adults with sickle cell disease | N = 99 | 5 to 25 years | Deficiency: <10ng/mL | 12% <10 ng/mL |
| Osunkwo et al. 2011^47^ | Vitamin D deficiency and chronic pain in sickle cell disease | N = 53 | 1 to 19 years | Profound  deficiency: <5 ng/mL  Severe  deficiency: 6 to 11 ng/mL  Deficiency: 12 to 20 ng/mL  Insufficiency: 12 to 20 ng/mL | 13% < 5 ng/mL  40% 6 to 11 ng/mL  32% 12 to 20 ng/mL  15% 12 to 20 ng/mL |
| Osunkwo et al. 2012^48^ | High dose vitamin D therapy for chronic pain in children and adolescents with sickle cell disease: results of a randomized trial | N = 39 | 7 to 21 years | Deficiency: <20 ng/mL  Insufficiency: <30 ng/mL | 52.5% <20 ng/mL  82.5% < 30ng/mL |
| Ozen et al. 2013^50^ | Frequency and risk factors of endocrine complications in Turkish children and adolescents with sickle cell disease | n = 50 | 4 to 18 years | Deficiency: <20 ng/mL  Insufficiency: 21 to 30 ng/mL | 63.1% <20 ng/mL  18.4% 21 to 30 ng/mL |
| Rovner et al. 2008^49^ | High risk of vitamin D deficiency in children with sickle cell disease | N = 61 | 5 to 18 years | Deficiency: <11 ng/mL  Insufficiency: 11 to <30 ng/mL | 33% <11 ng/mL |
| Sadat-Ali et al. 2011^53^ | Vitamin D level among patients with sickle cell anemia and its influence on bone mass | N = 186 | 18 and older | Deficiency: <20ng/mL  Insufficiency: 21 to 2 ng/mL | 82% <20 ng/mL |
| Van Der Djis et al. 1997^51^ | Serum Calcium and vitamin D status of patients wth sickle cell disease in Curaçao | N = 18 | 3 to 19 | Deficiency: < 10ng/mL | 0% < 10 ng/mL |
